# Prognostic role of red blood cell distribution width in patients with sepsis: a systematic review and meta-analysis

**DOI:** 10.1186/s12865-020-00369-6

**Published:** 2020-07-06

**Authors:** Lin Zhang, Cui-hua Yu, Kuan-peng Guo, Cai-zhi Huang, Li-ya Mo

**Affiliations:** 1 Department of clinical laboratory, Hunan children’s hospital, Changsha, China; 2 Department of GCP certified sites, The third hospital of Changsha City, Changsha, Hunan Province China

**Keywords:** Red cell distribution width, Sepsis, Septic shock, Mortality, Meta-analysis

## Abstract

**Background:**

Outcome prediction for patients with sepsis may be conductive to early aggressive interventions. Numerous biomarkers and multiple scoring systems have been utilized in predicting outcomes, however, these tools were either expensive or inconvenient.

We performed a meta-analysis to evaluate the prognostic role of red blood cell distribution width (RDW) in patients with sepsis.

**Methods:**

The online databases of Embase, Web of science, Pubmed, Corchrane library, Chinese Wanfang database, CNKI database were systematically searched from the inception dates to June, 24th, 2020, using the keywords red cell distribution width and sepsis. The odds ratio (OR) or Hazards ratio (HR) with corresponding 95% confidence intervals (95%CI) were pooled to evaluate the association between baseline RDW and sepsis. A random-effects model was used to pool the data, and statistical heterogeneity between studies was evaluated using the *I*^*2*^ statistic. Sensitivity and subgroup analyses were performed to detect the publication bias and origin of heterogeneity.

**Results:**

Eleven studies with 17,961 patients with sepsis were included in the meta-analysis. The pooled analyses indicated that increased baseline RDW was associated with mortality (HR = 1.14, 95%CI 1.09–1.20, Z = 5.78, *P* < 0.001) with significant heterogeneity (*I*^*2*^ = 80%, *P*_heterogeneity_ < 0.001). Similar results were found in the subgroup analysis stratified by site of infection, comorbidity, Newcastle-Ottawa Scale (NOS) score, study design, patients’ country. The predefined subgroup analysis showed that NOS score may be the origin of heterogeneity.

**Conclusions:**

For patients with sepsis, baseline RDW may be a useful predictor of mortality, patients with increased RDW are more likely to have higher mortality.

## Background

Despite the modern and advanced diagnostic methods, broad-spectrum antibiotics, and intensive care, sepsis mortality is still unacceptably high [1]. The prediction of outcome for patients with sepsis may be conductive to early aggressive interventions [2]. Numerous biomarkers and multiple scoring systems have been utilized to predict the outcome for patients with sepsis [3–6], however, these tools were either expensive or inconvenient.

Red cell distribution width (RDW) is an erythrocyte index, reflecting the heterogeneity in the size of circulating erythrocytes, which is used in diagnosis or differential diagnosis of hematological disease [7, 8]. Recently, RDW has proved to be a powerful predictor of outcome in many pathological conditions, including acute or chronic heart failure [9], acute pancreatitis [10], sepsis [11, 12], acute pulmonary embolism [13], acute kidney injury [14], etc. Although, the relevant mechanisms involved in the relationship of RDW and sepsis are not well clarified, it is critical to define the prognostic role of RDW in patients with sepsis. Given the inconsistent conclusions regarding the relationship of RDW and sepsis, we performed a comprehensive meta-analysis to evaluate the prognostic role of RDW in patients with sepsis.

## Methods

### Search strategies

Two reviewers (LZ and ChY) systematically and independently searched the online databases of Embase, Web of science, Pubmed, Corchrane library, Chinese Wanfang database, CNKI database from the inception dates to June, 24th, 2020, using the keywords sepsis, severe sepsis, septic shock and red blood cell distribution width to identify published articles evaluating the association between sepsis and RDW with no language restrictions.

### Selection criteria

Studies were selected based on the inclusion criteria: (1) The study design was a randomized clinical trial (RCT), a retrospective cohort study (RCS), a prospective cohort study (PCS) or a case control study (CCS); (2) A study with baseline RDW and clearly noted clinical outcomes during follow-up of sepsis patients; (3) A study with a reported hazard ratio (HR) or odds ratio (OR) and a corresponding 95% confidence interval (95%CI),or a study with no directly reported HR and 95%CI that we could reconstruct with *P* values and other reported data for RDW levels and mortality in sepsis patients. Individual case reports, abstracts, letters, editorials and review articles were excluded, as were studies without specific data concerning sepsis or RDW. When multiple published reports concerning the same cohort, we used only the first publication.

### Data extraction

Two investigators (LZ and ChY) independently screened the studies’ abstracts and titles, blinded to the authors and the journal titles, to identify all potential eligible studies. Potentially relevant articles were retrieved as full text and assessed for consistence with the inclusion criteria. Any uncertainties or discrepancies were discussed with other researchers and finally resolved by Li-ya Mo. We extracted the necessary data elements, including the first author’s last name, year of publication, country of the population, study design, sample size, gender ratio, mean age, cutoff value of RDW to define “elevated RDW”, clinical outcome, etc. The Newcastle-Ottawa Scale (NOS) was used to assess the quality of the included studies. The scale uses a star system (with a maximum of 9 stars) in three domains: selection of participants, comparability of study groups, and ascertainment of outcome or exposure. A Study with a scores of ≥7 was defined as high-quality study.

### Statistical analysis

Pooled effect sizes were reported as the HR with 95%CI, directly obtained from the original manuscript or calculated by other data reported in the manuscript. A random-effects model was performed to pool the data, and statistical heterogeneity between studies was evaluated using the *I*^*2*^ statistic. A *I*^*2*^ > 50% indicates significant statistical heterogeneity, Subgroup analysis was performed to explore the source of heterogeneity according to site of infection, comorbidity, NOS score, study design, patients ‘country. Sensitivity analysis was performed by deleting one study each time to evaluate the pooled effect. We evaluated the publication bias by examining funnel plots when the number of studies reporting the primary clinical outcomes was 10 or more. All meta-analyses were carried out using Revman version 5.3(provided by Cochrane Collaboration). All tests were dual-tailed, and *p* < 0.05 was defined as statistically significant.

## Results

### Studies retrieved and characteristics

**Fig. 1:**
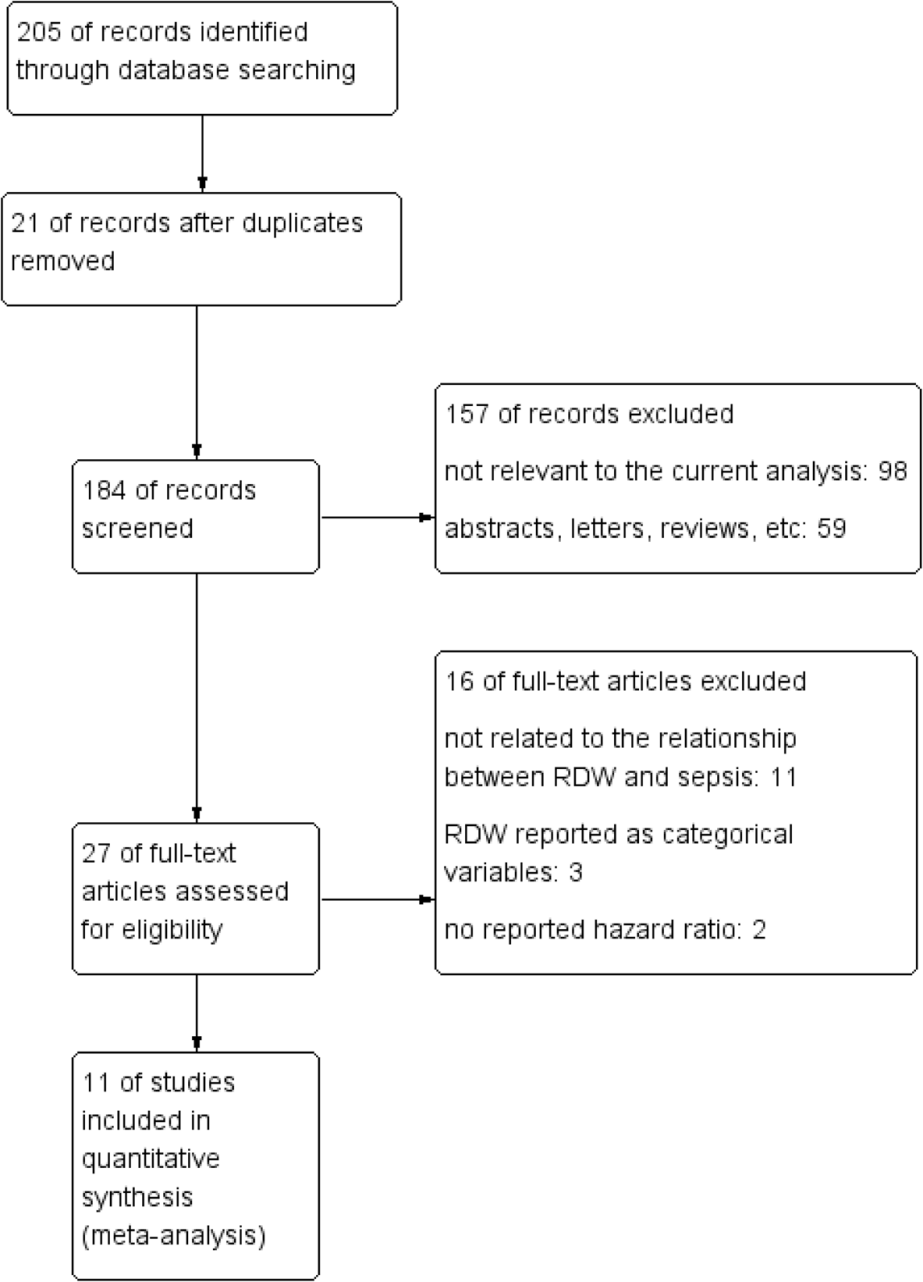
Flow diagram of selecting the literature and screening process

A flow diagram of data retrieval and study selection is shown in Fig. 1. A total of 205 potentially eligible studies were identified according to the inclusion criteria. Twenty-one duplicate records were discarded. The titles and abstracts of the remaining 184 records were then screened for inclusion, 157 records were discarded, either because they were not relevant to the current analysis or they were just abstracts, letters or reviews. Finally, the full texts of 27 studies were read, and 11 met the inclusion criteria.

Eleven studies with a total of 17,961 patients were included in the meta-analysis. The main characteristics of the included studies are presented in Table 1. The studies were conducted in Asia, Europe, North America, between 2014 and 2019. Two studies were prospective cohort studies, three were retrospective cohort studies, and the other six were case-control studies. The median age of the patients ranged from 59.5 to 81.5 years old. The proportion of males in the studies ranged from 50.5 to 67.7%. The patients’ primary comorbidities were hypertension or diabetes mellitus in eight studies [12, 15–18, 21–23], and the primary resource of infection were respiratory system in seven studies [11, 12, 17, 18, 20, 21, 23]. Patients in three studies with special type of sepsis were acute kidney injury undergoing continuous renal replacement therapy [15], community-acquired intra-abdominal sepsis [19] and cancer [20]. The primary clinical outcome was 28-day or 30-day mortality, hospital mortality, or all-cause mortality. The Cut-off of RDW ranged from 14.0 to 16.0%.

**Table 1:** Characteristics of 11 studies included in the meta-analysis

Study	Year	Country	design	Men/Patients	Age	Cut-off	Outcome
Cho [15]	2018	Korea	CCS	214/340	68 ± 13	14.8	28d mortality
Han [16]	2018	China	RCS	2220/4264	68 (54,79)	NM	all-cause mortality
Kim [17]	2015	Korea	CCS	242/458	78.0 (73.8,83.0)	15.0	30d mortality
Lorente [18]	2014	Spain	PCS	201/297	59.5 ± 16.5	14.5	30d mortality
Ozdogan [19]	2015	Turkey	CCS	55/103	64 ± 14	16.0	hospital mortality
Wang [12]	2018	China	RCS	70/117	81.5 ± 8.3	14.5	hospital mortality
Ding [20]	2018	China	CCS	50/78	65.7 ± 11.5	14.0	28d mortality
Gong [21]	2017	China	RCS	121/196	60.88 ± 18.11	15.0	28d mortality
Li [22]	2019	USA	CCS	6626/11691	67.1 ± 17.1	NM	28d mortality
Shen [11]	2016	China	CCS	103/204	81.5 ± 7.2	14.18	30d mortality
Wang [23]	2015	China	PCS	131/213	71.2 ± 16.8	15.0	28d mortality

Abbreviation: *PCS* Prospective cohort study; *RCS* Retrospective cohort study; *CCS* Case control study; *NM* Not mentioned

### NOS score

As is shown in Table 2, the Newcastle-Ottawa Scale (NOS) score of 11 studies ranged from five to eight, the NOS score of four studies were below seven.

**Table 2:** NOS score of 11 studies included in the meta-analysis

Study	Year	Selection	Comparability	Outcome/Exposure	Score
Cho [15]	2018	★★★	★	★★★	7
Han [16]	2018	★★★★		★★★	7
Kim [17]	2015	★★★	★	★★★	7
Lorente [18]	2014	★★★		★★	5
Ozdogan [19]	2015	★★★	★	★★★	7
Wang [12]	2018	★★★	★★	★★★	8
Ding [20]	2018	★★	★	★★★	6
Gong [21]	2017	★★★★	★	★★★	8
Li [22]	2019	★★★		★★★	6
Shen [11]	2016	★★★	★	★★	6
Wang [23]	2015	★★★★	★	★★★	8

### Association between RDW and outcome of patients with sepsis

**Fig. 2:**
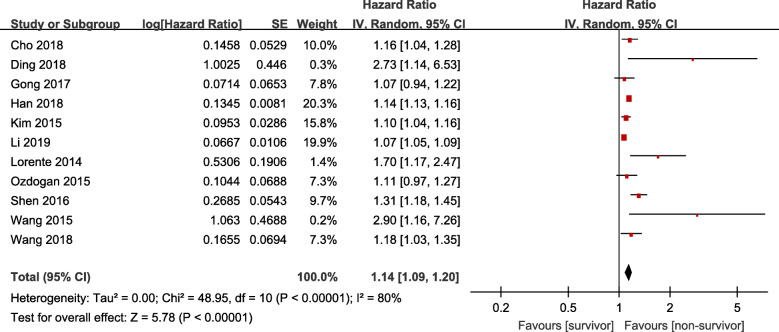
Meta-analysis results of RDW and clinical outcome in patients with sepsis

Eleven studies in the meta-analysis examined the association between RDW and patients’ mortality. RDWs in all 11 studies were reported as continuous variables. As shown in Fig. 2, the combined results of 11 studies showed that elevated RDW was associated with mortality (HR = 1.14, 95%CI 1.09–1.20, Z = 5.78, *P* < 0.001) with significant heterogeneity (*I*^*2*^ = 80%, *P*_heterogeneity_ < 0.001), that meant for patients with sepsis, each 1% increase in RDW, the risk of mortality increased by 14%.

### Sensitivity and subgroup analysis

**Fig. 3:**
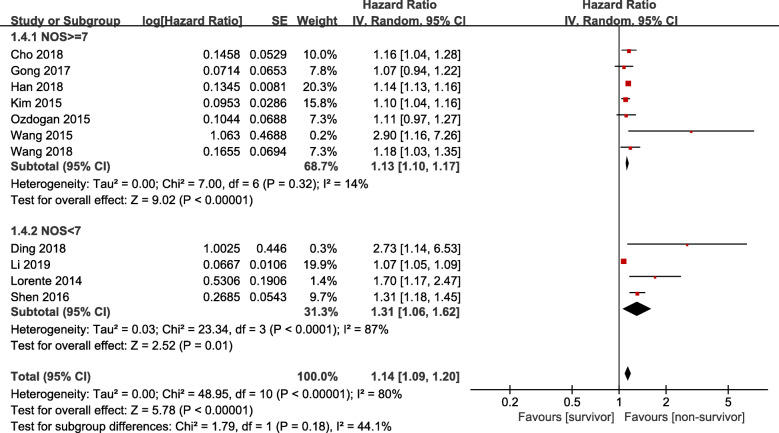
NOS subgroup analysis of RDW and clinical outcome in patients with sepsis

A single literature involved in the meta-analysis was deleted each time to detect the influence of the individual data set on the pooled HRs. There were no significant influences on heterogeneity across studies except the study by Li [22], the heterogeneity decreased from 80 to 58%. We also performed a predefined subgroup analysis according to study design (prospective cohort study, retrospective cohort study, or case control study), comorbidity, site of infection, NOS score (≥7 or < 7). We found that study design, comorbidity and site of infection didn’t influence the overall heterogeneity, while we found that there was lower heterogeneity (*I*^*2*^ = 14%) between studies of NOS ≥7, the NOS score may be the origin of heterogeneity. As is shown in Fig. 3, When the combined results of 7 studies with NOS ≥7, elevated RDW was also associated with higher mortality (HR = 1.13, 95%CI 1.10–1.17, Z = 9.02, *P* < 0.001) with lower heterogeneity (*I*^*2*^ = 14%, P _heterogeneity_ = 0.32). Furthermore, similar results were found in subgroup analysis stratified by site of infections, comorbidity, study design and region of patients, which were shown in Table 3.

**Table 3:** Subgroup analysis of association between RDW and clinical outcome

Variable	No. of trails	HR	95%CI		*P* value	Heterogeneity(I^2^)
Site of infection						
respiratory tract	6	1.15	1.10	1.20	< 0.001	71%
others	3	1.15	1.06	1.25	< 0.001	50%
Comorbidity						
hypertension and DM	8	1.12	1.10	1.13	< 0.001	81%
others	3	1.24	1.14	1.34	< 0.001	70%
NOS score						
≥ 7	7	1.13	1.10	1.17	< 0.001	14%
< 7	4	1.31	1.06	1.62	< 0.001	87%
Study design						
CCS	6	1.08	1.06	1.10	< 0.001	75%
RCS	3	1.14	1.13	1.16	< 0.001	0%
PCS	2	1.83	1.30	2.59	< 0.001	10%
Region						
Europe or North America	2	1.07	1.05	1.09	< 0.001	83%
Asia	9	1.14	1.13	1.16	< 0.001	53%

### Publication bias

**Fig. 4:**
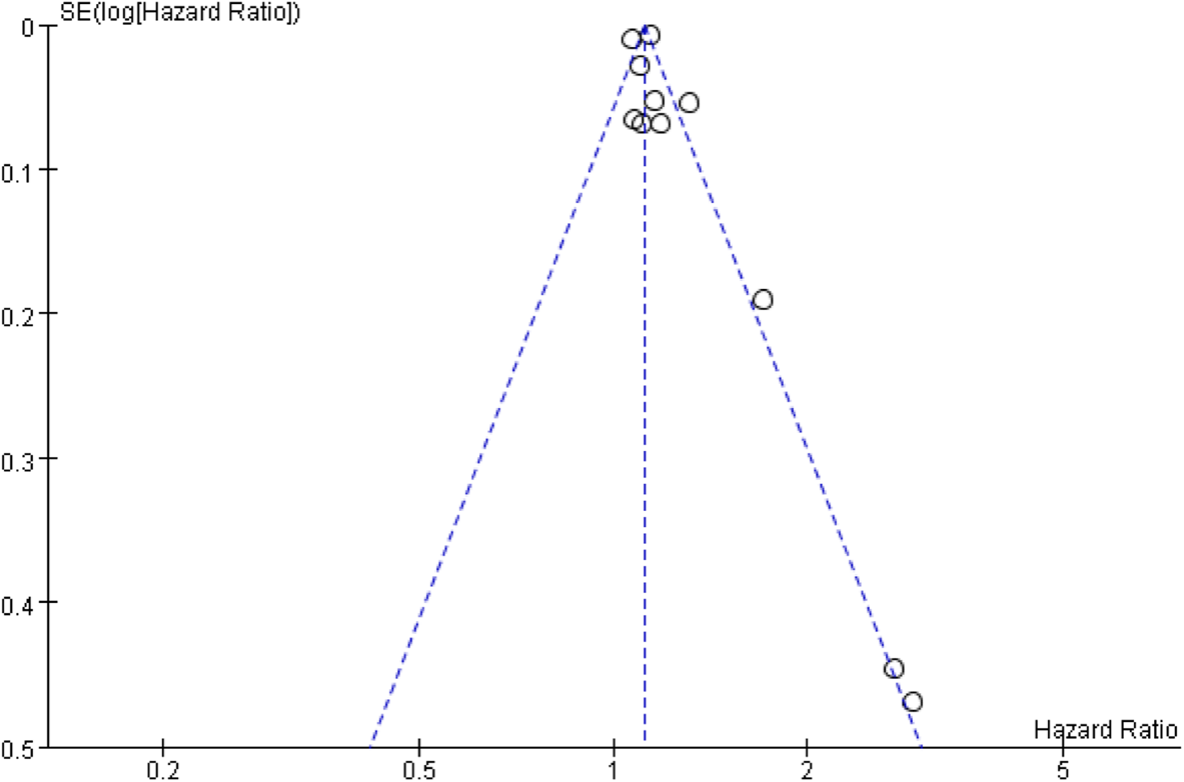
Funnel plot for the association between RDW and outcome in patients with sepsis

To evaluate the publication bias in the study, the included studies were conducted by funnel plot, which was not symmetrical, indicating the potential existence of publication bias (Fig. 4).

## Discussion

Numerous studies have reported the association between RDW and adverse clinical outcomes in various diseases, especially for mortality [9, 12]. This meta-analysis is the first time to combine the results of eligible studies concerning the association between RDW and sepsis. In the comprehensive meta-analysis including 17,961 sepsis patients from 11 studies, RDW was a remarkably useful predictor of mortality in patients with sepsis.

Also, there were other meta-analysis concerning the prognostic value of RDW in hematological malignancies [24], heart failure [25] or chronic kidney disease [26], etc. The previous researchers drew a similar conclusion as the ongoing meta-analysis: RDW may serve as a useful predictor of clinical outcome. In nine of the enrolled studies, baseline RDW significantly predicted the short-term (28 or 30 day) or long-term mortality (4 years), only two studies [19, 21] did not support the prognostic role of RDW in patients with sepsis. Ozdogan [19] recruited patients of a special subgroup of sepsis: the community-acquired intra-abdominal sepsis, and the primary clinical outcome was hospital mortality less than 15 days. While Gong [21] explored the role of baseline RDW and increasing of RDW in the whole course of disease, only the increasing of RDW but not the baseline RDW could predict the 28-day mortality.

The potential mechanisms underneath the association between increased RDW and higher risk of mortality remain largely unknown. Several latent mechanisms have been suggested to explain the reason why increased RDW leads to adverse outcome in sepsis patients. Firstly, An association between increasing RDW and elevated levels of acute phase reactants has been clearly demonstrated in previous researches [27], such as C-reactive protein (CRP), erythrocyte sedimentation rate (ESR), interlukin-6(IL-6), tumor necrosis factor (TNF) receptors I and II. This indicates that RDW may reflex the presence of inflammatory response, which can negatively affect the bone marrow function, iron metabolism and red blood cells homeostasis [28], then leading to blunted erythropoiesis (anisocytosis), an active role in the onset and progression of many human pathologies, and may also generate a negative impact on systemic inflammatory response syndrome [29] (sepsis). Secondly, high oxidative stress, one of the pathophysiologic entities of sepsis [30], can reduce RBCs survival [31] and increase the release of large premature RBCs into the peripheral circulation, which directly leading to elevated RDW. Thirdly, sepsis can alter glycoproteins and ion channels of the membrane in RBCs, which contributes to the change of RBCs morphology [32]. Lastly, RDW has proved to be associated with renal dysfunction, which is closely related with malnutrition and inflammation [33]. All above factors considered, it is reasonable to presume that elevated RDW may reflex an integrative measure of various harmful pathologic process, including oxidative stress, inflammatory response, renal dysfunction, malnutrition, which may occur simultaneously in sepsis.

Although there was significant heterogeneity across the included 11 studies, sensitivity analysis indicated that the pooled results were robust. After deleting one study each time, the pooled HRs remained stable. The study by Li [22] was retrospectively performed by collecting data from ICU patients in the USA, while the majority patients of the included studies were derived from Asia, racial or region differences may have effect on the significant heterogeneity. We also performed the predefined subgroup analysis, and found that NOS score may be the origin of heterogeneity, NOS score of four studies was below seven. They were designed retrospectively, the primary and other latent confounding factors were not strictly controlled, leading to limited comparability. Similar results were found in subgroup analysis stratified by site of infection, comorbidity, study design and country of patients.

This meta-analysis does have some limitations that should call for cautious interpretation of the results. First, the majority of the included studies were conducted in Asia, which could not be representative of other areas. The application of prognostic role of RDW in sepsis in other areas needs further study in advance. Second, different RDW cutoffs, NOS score and sample size across the 11 studies may attribute to the heterogeneity, and cause bias in the meta-analysis. Third, despite adjusting for several potential confounding factors and prevalent conditions, it is possible that there might be residual confounding from diseases and medications not included in the current study. Fourth, we did not obtain the data about iron, folate, vitamin B_12_, erythropoietin or reticulocyte count, which may affect the RDW level [34]. Fifth, unsymmetrical funnel plot indicated the existence of publication bias, some negative results might be unpublished.

## Conclusions

Red cell distribution width at baseline is associated with mortality of patients with sepsis, RDW may be a simple and useful prognostic marker for the patients with sepsis. This study provides support for further research of adding RDW to other established outcome predicting systems and markers of mortality and inflammation in patients with sepsis.

## Data Availability

All data analyzed during the study are included in this published article (and its supplementary information files) and are available from the included studies, which are fully referenced.

## Abbreviations

RDW: Red blood cell distribution width
OR: Odds ratio
HR: Hazards ratio
95%CI: 95% confidence intervals
RCT: Randomized clinical trial
RCS: Retrospective cohort study
PCS: Prospective cohort study
CCS: Case control study
NOS: Newcastle-Ottawa scale
NM: Not mentioned
CRP: C-reactive protein
ESR: Erythrocyte sedimentation rate
IL-6: Interlukin-6
TNF: Tumor necrosis factor
RBC: Red blood cell
ICU: Intensive care unit
